# Longitudinal Assessment of Body Composition and Inflammatory Status in Rheumatoid Arthritis During TNF Inhibitor Treatment: A Pilot Study

**DOI:** 10.3390/ijms26157635

**Published:** 2025-08-07

**Authors:** Natalia Mena-Vázquez, Aimara García-Studer, Fernando Ortiz-Márquez, Sara Manrique-Arija, Arkaitz Mucientes, Jose Manuel Lisbona-Montañez, Paula Borregón-Garrido, Patricia Ruiz-Limón, Rocío Redondo-Rodriguez, Laura Cano-García, Antonio Fernández-Nebro

**Affiliations:** 1 Instituto de Investigación Biomédica de Málaga y Plataforma en Nanomedicina-IBIMA Plataforma BIONAND, 29590 Málaga, Spain; aimara.garcia.sspa@juntadeandalucia.es (A.G.-S.); fernando.ortiz.sspa@juntadeandalucia.es (F.O.-M.); sara.manrique.sspa@juntadeandalucia.es (S.M.-A.); arkaitz.mucientes@ibima.eu (A.M.); jose.lisbona@ibima.eu (J.M.L.-M.); paula.borregon.sspa@juntadeandalucia.es (P.B.-G.); patriciaruizlimon@ibima.eu (P.R.-L.); rocio.redondo.sspa@juntadeandalucia.es (R.R.-R.); laura.cano.sspa@juntadeandalucia.es (L.C.-G.); afernandezn@uma.es (A.F.-N.); 2 UGC de Reumatología, Hospital Regional Universitario de Málaga, 29009 Málaga, Spain; 3 Departamento de Medicina, Universidad de Málaga, 29071 Málaga, Spain; 4 Unidad de Gestión Clínica de Endocrinología y Nutrición, Hospital Clínico Virgen de la Victoria, 29010 Málaga, Spain; 5 CIBER Fisiopatología de la Obesidad y Nutrición (CIBEROBN), Instituto de Salud Carlos III, 28029 Madrid, Spain

**Keywords:** rheumatoid arthritis, body composition, TNF inhibitors, sarcopenia, inflammation, inflammatory activity, cytokines

## Abstract

Rheumatoid arthritis (RA) is a chronic inflammatory disease frequently associated with alterations in body composition, including reduced lean mass and increased fat mass. These alterations are thought to be driven by persistent systemic inflammation, which may be influenced by inflammatory activity and by therapeutic interventions. Objectives: This pilot study aimed to provide preliminary data on changes in body composition and inflammatory activity in biologic-naive patients with active RA during the initial 6 months of TNF inhibitor treatment, and to compare baseline body composition with healthy controls. We conducted a single-center, observational, 24-week pilot study of 70 biologic-naive RA patients with moderate-to-severe disease activity and 70 matched healthy controls. Lean mass, fat mass, and lean mass index (LMI) were measured using dual-energy X-ray absorptiometry at baseline for both groups, and after 6 months only in the RA group. Clinical, laboratory, adipokines, and cytokine parameters were also recorded. At baseline, RA patients had lower lean mass and LMI than controls. Over 6 months, RA patients showed significant clinical and laboratory improvement, with a corresponding increase in lean mass and LMI. No statistically significant change was observed in fat mass. The increase in lean mass was paralleled by a reduction in inflammatory markers. The LMI was inversely associated with female sex (β = −0.562) and C-reactive protein (β = −0.432) and directly associated with body mass index (β = 0.570). Similar associations were observed for total lean mass and change in lean mass, as well as for DAS28 (β = −0.333). This pilot study provides preliminary evidence that TNF inhibitor therapy may be associated with increased lean mass and decreased inflammation in RA patients. Owing to the absence of a comparator RA group not receiving TNF inhibitors, these findings should be interpreted as hypothesis-generating.

## 1. Introduction

Rheumatoid arthritis (RA) is a chronic immune-mediated inflammatory disease that affects around 1% of the world’s population [1,2]. The etiology–pathogenesis of RA is complex, characterized by overproduction of pro-inflammatory cytokines and autoantibodies, which leads to chronic inflammation and joint damage [3,4]. However, chronic inflammation not only causes joint damage but can also affect all organ systems, making RA a systemic disease. As for the inflammatory component, RA patients have been observed to be at a significantly increased risk of cardiovascular disease, which is one of the main causes of death [4,5]. Chronic inflammation associated with RA can also affect body composition by altering the distribution of lean and fat mass, leading, in turn, to more severe inflammation, increased cardiovascular risk, and an increase in morbidity and mortality in affected patients [3,6].

RA patients have a significantly increased body mass index (BMI) and total fat mass, especially around the trunk, indicating a change in body composition and distribution [7,8]. Furthermore, this increase in total fat mass has been associated with greater disease activity and poorer physical functioning in patients with RA [9,10]. Similarly, between 20% and 40% of RA patients experience a reduction in their muscle mass, muscle weakness, and sarcopenia. According to the diagnostic criteria of the European Working Group on Sarcopenia in Older Persons (EWGSOP), these findings constitute sarcopenic obesity [11,12,13]. The changes observed have been associated with cytokine-mediated systemic inflammation, reduced physical activity [14], and a greater risk of malnutrition than in the general population [15,16]. It is essential to determine how treatment can interrupt this spiral of severity and improve patients’ general health.

The effect of anti-tumor necrosis factor (TNF) therapy on body composition in RA has been associated with inconsistent results across studies [17,18,19,20]. While some have reported increased fat mass without changes in muscle mass [19,20], others, such as Hasegawa et al. [18], found improvements in lean mass and reduced sarcopenia. A recent meta-analysis [20] also reported a lack of significant overall effect on total lean mass. These discrepancies may be due to differences in study design, populations, treatment duration, and methods used to assess muscle mass. Studies based on small patient populations show increased fat mass after 6–12 months of therapy with TNF inhibitors in RA, although no effect on muscle mass was reported [21,22,23]. Notwithstanding, in their study of 48 patients with RA, Hasegawa et al. [18] found an improvement in total lean mass and a reduced percentage of patients with sarcopenia after 12 months’ therapy with TNF inhibitors. Similarly, Tournadre et al. [24] reported increased muscle mass in 21 patients with active RA after 6 and 12 months of treatment with tocilizumab. However, most of these studies lacked a control group, patients were not biologic-naive, and other metabolic factors (e.g., inflammatory cytokines and the average inflammatory activity during the course of the disease) were not analyzed.

In summary, while there is evidence of the beneficial effects of TNF inhibitors on the body composition of people with RA, further research is necessary to better clarify these effects and their clinical relevance. In the present study, we propose to fill this gap in our knowledge through a prospective evaluation of the impact of TNF inhibitors on the body composition of patients with RA and marked inflammatory activity. The primary aim of this pilot study was to provide preliminary data on changes in body composition and inflammatory activity during the first 6 months of TNF inhibitor treatment in biologic-naive patients with active RA. Secondary objectives were to describe baseline fat and lean mass in these patients compared to healthy controls, and to explore clinical and laboratory factors associated with body composition parameters in RA.

Due to the absence of a comparator group of RA patients not receiving TNF inhibitors, the longitudinal findings should be considered exploratory and hypothesis-generating.

## 2. Results

### 2.1. Baseline Characteristics of the Sample

The study sample comprised 140 participants: 70 patients with RA and 70 healthy controls. Table 1 shows the baseline characteristics of both groups. No significant differences were recorded with respect to sex, age, or race, with a female predominance in both groups (81%) and a mean age of 56 years. However, major differences were detected for comorbid conditions and cardiovascular risk factors. Compared with the controls, patients with RA more frequently had an associated comorbid condition (100% vs. 64.3%; *p* < 0.001) or multiple comorbid conditions (54.3% vs. 31.7%; *p* = 0.042), as well as a higher median number of comorbid conditions (*p* = 0.021), Charlson Comorbidity Index (*p* < 0.001), and age-adjusted Charlson Comorbidity Index (*p* < 0.001). As for cardiovascular risk factors, a greater number (%) of patients with RA had a history of smoking than controls (*p* = 0.037).

Most patients had seropositive disease (80%), with erosions in half of the cases and established disease (median [IQR] disease duration, 126.4 months [34.6–184.8]). At T0, all patients had a DAS28-CRP score indicating moderate or high inflammatory activity (mean [SD], 4.9 [1.1]). All RA patients were receiving csDMARDs, and 74% were taking corticosteroids at T0. Among the bDMARDs started at T0, most (87.1%) were with a biosimilar of adalimumab while 12% were with a biosimilar of etanercept.

While no significant differences between patients and controls were recorded for anthropometric values, such as weight, height, and BMI, the mean (SD) total lean mass was significantly lower in the RA group than in the controls (38.0 [8.0] vs. 41.1 [7.7]; *p* = 0.012), as was the LMI (14.4 [2.3] vs. 15.9 [2.1]; *p* < 0.001) (Table 2). Similarly, higher prevalence values were detected in patients than in controls for sarcopenia (28.6% vs. 12.9%; *p* = 0.022) and sarcopenic obesity (10% vs. 1.4%; *p* = 0.029). However, physical activity (measured in METs) was greater among controls (median [IQR] = 990.0 [561.0–1993.5] vs. 594.0 [231.0–1386.0]; *p* = 0.033]).

As for the inflammatory cytokine and adipokines profile, values were higher in patients than in controls for adiponectin (*p* = 0.002), IL-6 (*p* < 0.001), CRP (*p* < 0.001), and LDL-oxidase (*p* < 0.001), as well as for other laboratory parameters such as ESR (*p* < 0.001) and homocysteine (*p* = 0.045). Patients had lower levels of hemoglobin (*p* = 0.011).

### 2.2. Study of Body Composition, Adipokines, and Inflammatory Factors at Baseline and at 6 Months in Patients with RA Treated with TNF Inhibitors

**Figure 1:**
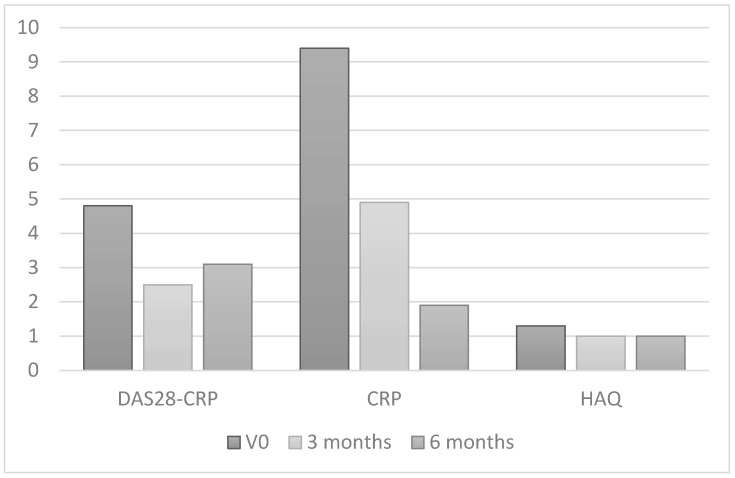
Changes in inflammatory activity and physical functioning in patients with RA after 6 months of treatment with TNF inhibitors.

Table 3 shows clinical and inflammation-related characteristics both at baseline and at 6 months of treatment with TNF inhibitors. Patients experienced a significant clinical improvement in disease activity according to the DAS28-CRP (*p* < 0.001) and in a series of parameters that make up disease activity indices, such as the tender joint count (*p* < 0.001), the swollen joint count (*p* < 0.001), the mean pain score (*p* < 0.001), the patient global assessment (*p* < 0.001), and the physician global assessment (*p* < 0.001). Physical functioning according to the HAQ also improved in this group (*p* < 0.001) (Figure 1).

As for body composition, at 6 months of treatment with TNF inhibitors patients experienced a significant increase in total lean mass (*p* < 0.001) and in lean mass in the arms (*p* < 0.001), trunk (*p* = 0.003), and gynoid area (*p* = 0.031). They also experienced a significant increase in LMI (*p* = 0.016). The percentage of patients with sarcopenia was also lower than in controls (28.6% vs. 11.4%; *p* = 0.004). Fat mass, however, decreased slightly during treatment, although the difference was not statistically significant (2.8 [0.9] vs. 2.6 [0.9]; *p* = 0.121).

As for cytokines and adipokines, a significant decrease was observed for inflammatory cytokines, including IL-6 (*p* < 0.001), CRP (*p* < 0.001), IL-1β (*p*< 0.001), IGF-1 (*p* < 0.001), LDL-oxidase (*p* < 0.001), and ESR (*p* = 0.023). Hemoglobin levels increased (*p* = 0.029). Furthermore, during therapy a significant increase was recorded in values for resistin (*p* < 0.001) and adiponectins (*p* = 0.035) and a decrease in leptin values (*p* < 0.001).

### 2.3. Clinical and Laboratory Characteristics of RA Associated with Lean Mass

**Figure 2:**
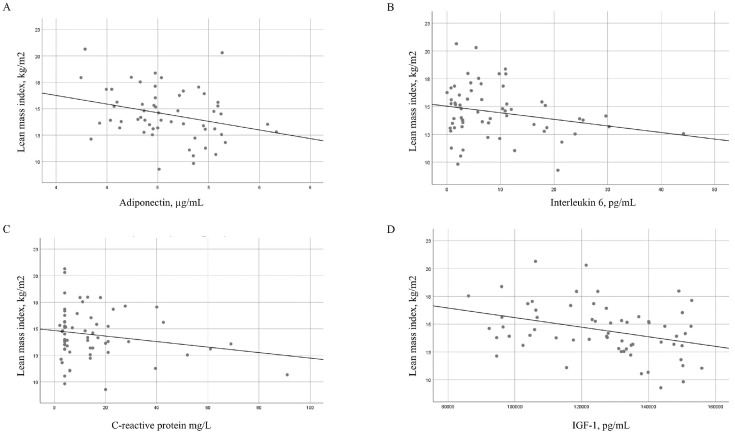
Regression slopes for lean mass index with adiponectin and cytokines in patients with RA. Regression slopes for the lean mass index (kg/m^2^) and (**A**) adiponectin levels (µg/mL), (**B**) interleukin 6 levels (pg/mL), (**C**) C-reactive protein levels (mg/L), and (**D**) insulin-like growth factor 1 levels (pg/mL).

Table 4 shows the bivariate correlations between LMI, baseline lean mass, and the change in lean mass after 6 months of treatment with TNF inhibitors in patients with RA according to clinical, laboratory, and inflammation-related variables. Significant inverse correlations were observed between LMI and values for adiponectin (*p* = 0.019), IL-6 (*p* = 0.001), CRP (*p* = 0.046), IGF-1 (*p* = 0.009), and ESR (*p* = 0.010). Lean mass was also significantly inversely correlated with the values for adiponectin (*p* = 0.019), IL-6 (*p* = 0.024), and IGF-1 (*p* = 0.002) (Figure 2). BMI was directly associated with LMI (*p* < 0.001) and total lean mass (*p* = 0.013). Moreover, the change in lean mass at 6 months of treatment with TNF inhibitors was significantly and inversely correlated with disease duration (*p* = 0.004), inflammatory activity by DAS28-CRP at the cut-off (*p* = 0.023), cumulative DAS28-CRP (*p* = 0.011), and CRP (*p* = 0.012), as well as with physical functioning according to the HAQ at the cut-off (*p* = 0.024) and cumulative HAQ (*p* = 0.015).

### 2.4. Multivariate Analysis

Table 5 shows the results of the univariate and multivariate analyses for lean tissue in patients with RA. The dependent variables in the three models were LMI, total lean mass (in kg), and change in lean mass. The variables evaluated to determine their role as predictors were sex, age, cumulative DAS28-CRP, cumulative HAQ, laboratory values (CRP, IGF-1, adiponectin, and leptin), and physical activity by IPAQ (METs).

As can been seen in Table 5, after adjustment for the remaining variables, CRP levels were associated with the three measures of lean mass in patients with RA. The LMI was inversely associated with female sex (β = −0.562) and CRP (β = −0.432) and directly associated with BMI (β = 0.570). Similarly, in the case of total lean mass, significant associations were found with female sex (β = −0.670) and CRP (β = −0.340), and a direct association was found with BMI (β = 0.560). Finally, the change in lean mass was negatively associated with inflammation-related parameters, such as CRP (β = −0.440) and DAS28 (β = −0.333).

## 3. Discussion

Increasing evidence links inflammatory activity with changes in body composition among patients with RA. The main findings are increased fat mass and reduced lean mass [11,25,26]. Therefore, we performed a study to compare body composition between patients with active RA and healthy controls before starting therapy with biologics to analyze changes in inflammatory activity and body composition after 6 months of treatment with TNF inhibitors.

In this context, we found that patients with active RA and an inadequate response to csDMARDs had a lower total lean mass and LMI at baseline than healthy controls; no differences were found for parameters associated with fat mass. In this sense, several studies have reported that patients with RA have lower lean mass and greater sarcopenia than healthy controls, irrespective of the evaluation method used [27,28]. While the exact mechanisms underlying muscle loss in patients with RA are not completely clear, they are believed to be multifactorial and involve disease-related inflammatory activity, reduced mobility, and treatment with corticosteroids [29]. Consistent with these factors, compared with controls the patients with RA in the present study had higher baseline levels of cytokines and other inflammatory markers, were taking corticosteroids, and engaged less frequently in physical activity.

After 6 months treatment with TNF inhibitors, our analysis revealed a significant increase in total lean mass, LMI, and lean mass on the arms, trunk, and gynoid area, as well as a reduction in the percentage of patients with sarcopenia. These findings are consistent with those of other studies, such as that of Hasegawa et al. [18], who analyzed 48 patients with RA and found an improvement in total lean mass and a reduction in the percentage of patients with sarcopenia after 12 months of treatment with TNF inhibitors. Similarly, Tournadre et al. [24] found an increase in muscle mass in 21 patients with active RA after 6 and 12 months of treatment with tocilizumab. These authors associated the improvement in lean mass with reduced inflammatory activity and the possible effect of biologics on inflammatory cytokines, such as IL-6. However, in contrast to our findings, these authors did not compare lean mass between patients and controls, and patients were not biologic-naive. Similarly, they did not directly analyze inflammatory cytokine levels or average cumulative inflammatory burden throughout the course of the disease. In this sense, we were also able to show that patients with RA initially presented higher baseline levels of inflammatory parameters, cytokines, and adipokines than controls and that these parameters improved significantly after 6 months of therapy with TNF inhibitors. In fact, after 6 months of treatment a significant clinical improvement was observed in disease activity (according to the DAS28-CRP), tender and swollen joint count, evaluation of pain, and physical functioning (according to the HAQ). In addition to modulating inflammatory cytokines and Wnt signaling components—which are known to be altered in RA and involved in disease activity and bone resorption [30]—TNF inhibitor therapy can influence intracellular stress-response pathways in immune cells. Recent studies have demonstrated that effective anti-TNF treatment can restore the balance between autophagy and apoptosis in circulating immune cells, as evidenced by reduced LC3-II expression and increased apoptotic cell death in responders to treatment, correlating with clinical improvement [31]. This restoration of appropriate immune cell turnover may reduce synovial hyperplasia and overall inflammatory burden, potentially complementing the observed improvements in lean body mass and supporting additional mechanisms underlying therapeutic responses in RA.

Beyond these clinical and inflammatory improvements, we also observed relevant changes in adipokines and anabolic markers that may explain the observed increase in lean mass. Specifically, levels of resistin and adiponectin increased significantly, while leptin levels decreased. In parallel, levels of IGF-1, a key anabolic hormone, increased significantly during follow-up. These shifts suggest that TNF inhibitor therapy may contribute to restoring a more favorable metabolic profile [32]. Moreover, baseline LMI values correlated inversely with IL-6, CRP, IGF-1, ESR, and adiponectin values, thus reinforcing the link between systemic inflammation, metabolic imbalance, and muscle loss. While adiponectin is generally considered anti-inflammatory, in RA it can, paradoxically, promote IL-6 secretion and catabolic signaling [33]. Leptin may also exert pro-inflammatory effects in this context. Conversely, IGF-1 promotes protein synthesis and inhibits muscle degradation. Therefore, improvements in lean mass may result not only from the control of inflammation but also from the modulation of metabolic and anabolic pathways [34].

Furthermore, the present study evaluated factors associated with lean mass by measuring three associated variables: LMI, total lean mass, and the change (or increase) in lean mass. An independent association was observed between these three measures of lean mass and inflammatory activity, sex, and body mass, which were also associated with LMI and total lean mass.

The inverse association between lean mass and female sex could be explained by the various physiological and hormonal differences between men and women. This finding agrees with those of previous studies that have also reported a greater prevalence of sarcopenia in women when the criteria used were based on lean mass. However, the association between sex and the new EWGSOP2 criteria for sarcopenia [35], which include grip strength and physical performance, is not clear, suggesting that the difference in prevalence for sarcopenia described in some studies is due mainly to the greater prevalence of low muscle mass in women more than to low strength [36,37]. Thus, a recent systematic review and meta-analysis reported that, based on the new EWSGOP2 criteria, sarcopenia was more prevalent in men than in women (11% vs. 2%), whereas, based on the evaluation of the International Working Group on Sarcopenia [35], the prevalence was greater in women (17% vs. 12%) [37]. An association has also been reported between lean mass and BMI [17,38]. Muscle mass is closely related to general body size; in other words, a higher body mass may reflect greater body weight owing to the greater amount of muscle mass. Therefore, people with greater muscle mass may have a high BMI without necessarily having excess body fat [38].

We measured physical activity using MET units. Patients with RA and marked disease activity engaged less frequently in physical activity than controls, possibly because of the tenderness and swelling associated with disease burden. After 6 months of treatment with TNF inhibitors, median physical activity increased in patients from low (<600 METs) to moderate (>600 METs). However, the multivariate analysis did not show physical activity to be associated with the gain in lean mass. This increase in physical activity after 6 months of treatment may be related to improvement in pain and disease activity, although it may not be of sufficient intensity or duration to exert an effect on muscle mass without additional intervention through an exercise program.

Our study has both strengths and limitations. First, this is a pilot study without a comparator group of RA patients not treated with TNF inhibitors, which precludes drawing conclusions about the specific effect of therapy or differentiating outcomes from natural disease evolution or other confounders. The within-patient longitudinal analysis is hypothesis-generating and intended to inform the design of future controlled studies. The inclusion of healthy controls allowed us to describe baseline differences, but did not resolve the limitation with respect to causal inference. Longer-term follow-up and inclusion of a TNF inhibitor-naive cohort could reveal additional differences. A key strength is that we were able to demonstrate a change in lean mass and in inflammatory activity measured using various parameters after 6 months of treatment with TNF inhibitors. Moreover, the increase in lean mass was associated with reduced inflammatory activity in patients with RA. The 6-month follow-up period was chosen based on clinical practice standards and previous studies showing that improvements in lean mass can be observed within this timeframe [19]. However, a 6-month period may not be sufficient to capture changes in fat mass, which typically require longer observation periods.

## 4. Materials and Methods

### 4.1. Study Design, Data Source, and Sampling

We conducted a 24-week, single-center, prospective, observational, pilot longitudinal study in a cohort of biologic-naive patients with established RA initiating TNF inhibitor therapy. The decision to initiate TNF inhibitor therapy was made exclusively by the treating rheumatologist as part of routine clinical practice. The study was performed in the Department of Rheumatology of Hospital Regional Universitario de Málaga (HRUM), Malaga, Spain. The analyses were performed at the Instituto de Investigación Biomédica de Málaga-Plataforma BIONAND. The study was approved by the Research Ethics Committee of HRUM (code 03/2022 PI 12). All patients provided their written informed consent before participating in the study.

#### 4.1.1. Patients

Patients with RA fulfilling the inclusion criteria were recruited consecutively from the Rheumatology Clinic of HRUM between June 2022 and June 2023. The inclusion criteria were as follows: (1) RA diagnosed according to the 2010 criteria of the American College of Rheumatology/European League Against Rheumatism (ACR/EULAR) [39]; (2) age > 16 years; (3) inadequate response to conventional systemic disease-modifying antirheumatic drugs (csDMARDs); (4) initiation of the first biologic therapy with a TNF inhibitor; (5) moderate-to-high disease activity, defined as a 28-joint Disease Activity Score with C-reactive protein (DAS28-CRP) ≥ 3.2. The exclusion criteria were as follows: (1) rheumatic diseases other than RA (except secondary Sjögren’s syndrome); (2) any condition that could significantly affect body composition (e.g., uncontrolled thyroid disease, active cancer, advanced kidney/liver disease); (3) pregnancy; (4) inability to undergo dual-energy X-ray absorptiometry (DXA) due to physical or technical limitations.

#### 4.1.2. Controls

As with the cases, the controls had to be aged >16 years, although with no history of inflammatory disease. The controls were matched by age and sex with the cases. They were selected consecutively from the same social setting as the cases and had to fulfill the same exclusion criteria.

### 4.2. Study Protocol

#### 4.2.1. Baseline Evaluation of Participants

RA patients from the HRUM cohort are usually treated jointly by rheumatologists and specialist nurses according to a pre-established protocol for the collection of clinical, anthropometric, and laboratory data every 3 or 6 months, or more frequently if clinically necessary. After providing their written informed consent, all participants were specifically evaluated for this study. This evaluation was considered the baseline visit (T0), at which patients underwent an interview and a standardized physical examination that included joint assessment (tender and swollen joint count), vital signs, weight, height, BMI, and waist and hip circumferences.

Participants also completed a custom-designed questionnaire that collected data on sociodemographic information, disease duration, comorbidities, lifestyle factors (physical activity, diet), medication use, and prior clinical history relevant to RA. This questionnaire was developed by the research team to ensure the comprehensive collection of variables known to influence body composition and inflammatory activity.

Biological samples were obtained after a 12 to 16 h overnight fast (before 10:00 a.m.) to reduce variability due to circadian and nutritional influences. These samples were used for measuring inflammatory markers (CRP, erythrocyte sedimentation rate [ESR]), pro-inflammatory cytokines (TNF-α, IL-6, IL-1β), adipokines (leptin, resistin, adiponectin), IGF-1, and other standard laboratory parameters (hemoglobin, lipid profile). On the same day, patients also underwent densitometry to assess body composition using a DXA device (GE Lunar Prodigy enCORE™ 2006, GE Healthcare, Madison, WI, USA) following the manufacturer’s instructions. The equipment was calibrated using a standard lumbar spine phantom and operated at the highest resolution. Participants were scanned in the supine position. All clinical and laboratory data from cases and controls were entered into a study database for further analysis.

#### 4.2.2. Prospective Evaluation of Patients with RA

All patients were evaluated prospectively. Patients underwent a clinical evaluation and physical examination, and data were collected at 12 weeks (T1) and at 24 weeks (T2) after the baseline visit (T0). Biological samples were collected again at T2, and body composition was evaluated using full-body DXA under the same conditions as at T0. All the tests were performed at the same clinic and during the same timeframe as at T0 by the same staff.

### 4.3. Definition of Variables

#### 4.3.1. Anthropometric Variables and Body Composition

Body composition was assessed using DXA at T0 and T2 based mainly on the measurement of total lean mass and total fat mass. These measurements were complemented by assessment of fat and lean mass in specific regions of the body, including the trunk, arms, legs, and android and gynoid areas. The fat mass index was defined as fat mass (kg)/height squared (m^2^); the lean mass index (LMI) was defined as lean mass (kg)/height squared (m^2^). The delta value for total lean mass (kg) was calculated as the difference in total lean mass during the 6 months of treatment with TNF inhibitors. Sarcopenia was defined as a relative skeletal mass index <5.5 kg/m^2^ for women and <7.26 kg/m^2^ for men [11].

Other anthropometric variables included BMI, which was calculated as body weight (kg) divided by height squared (m^2^). Patients were distributed into 4 BMI groups according to the criteria of the World Health Organization (WHO) [40], as follows: (1) underweight (<18.5); (2) normal weight (18.5–24.9); (3) overweight (25–29.9); (4) obesity (≥30). Waist circumference (cm) and hip circumference (cm) were also recorded, and the waist–hip ratio was calculated [41].

#### 4.3.2. Cytokines and Inflammatory Activity

The degree of joint inflammatory activity, biomarkers of inflammation, and adipokines were assessed at T0 and T2. Inflammatory mediators such as TNF-α, IL-1β, and IL-6 were analyzed in plasma using Quantiglo enzyme-linked immunosorbent (ELISA) kits (R&D Systems Inc., Minneapolis, MN, USA) following the manufacturer’s instructions. Levels of adipocytokines (leptin, resistin, and adiponectin) and insulin-like growth factor 1 (IGF-I) were determined in patients with RA and controls using ELISA (Mediagnost GmbH, Tübingen, Germany).

We collected data on disease activity, including CRP (mg/L), ESR (mm/h), and disease activity itself using the DAS28 (on a continuous scale of 0 to 9.4), with activity classified as high–moderate (≥3.2) or low–remission (<3.2) [42]. We also used the Spanish version of the Health Assessment Questionnaire (HAQ) to evaluate physical functioning [43]. Inflammatory activity during the course of the disease (time from diagnosis to T0) was measured, and cumulative DAS28-CRP values were calculated.

#### 4.3.3. Remaining Variables

We also recorded epidemiological, clinical, and therapy-related variables. We recorded age, sex, and race and evaluated comorbid conditions using the Charlson Comorbidity Index and age-adjusted Charlson Comorbidity Index [44,45]. Multimorbidity was defined as the co-occurrence of 2 or more chronic diseases, in addition to RA [46]. Physical activity was measured in metabolic equivalent of task units (METs) using the International Physical Activity Questionnaire (IPAQ) [47], and adherence to the Mediterranean diet was evaluated using a validated questionnaire, which considered a score of >9 over 14 as good adherence [48]. As for the clinical variables, we recorded data for time since diagnosis (to the cut-off date) and diagnostic delay (time since onset of symptoms to diagnosis). We recorded the presence of a positive rheumatoid factor titer, which was defined as levels > 20 IU/mL, the presence of a positive anti-citrullinated peptide antibody, which was defined as levels > 10 IU/mL, and the presence of joint erosions. Other laboratory data assessed included the ESR (mm/h) and levels of hemoglobin (g/dL), total cholesterol (mg/dL), low-density lipoprotein (LDL) cholesterol (mg/dL), high-density lipoprotein cholesterol (mg/dL), and triglycerides (mg/dL).

The therapy-related data recorded were treatment with csDMARDs and type of TNF inhibitor at T0, as well as corticosteroids use and dose of corticosteroids.

### 4.4. Statistical Analysis

We performed a statistical analysis of the main clinical and laboratory variables. Qualitative variables were expressed as numbers and percentages, and quantitative variables were expressed as mean (SD) or median (IQR) depending on the normality of their distribution, as assessed using the Kolmogorov–Smirnov test. The χ^2^ test and *t* test or Mann–Whitney test were used to compare the main characteristics between patients and controls. Comparisons were made between associated variables at baseline and at 6 months of treatment using the Wilcoxon test. The main clinical characteristics of patients with RA were correlated with LMI, total lean mass, and the change in lean mass during the 6 months of treatment with TNF inhibitors. Finally, 3 multivariate linear regression models were constructed (dependent variables: LMI, total lean mass at T0, and change in lean mass during follow-up) to study the independent variables associated with lean mass in patients with RA. The variables selected for the multivariate analysis were those that proved to be significant in the bivariate analysis and those that were of clinical interest. Missing data were only present in certain laboratory variables (adiponectin: 2.8%; IL-6: 8.5%), owing to issues in sample handling. As these missing values were considered missing completely at random, they were imputed using multiple imputation by chained equations with the automatic imputation procedure in SPSS. Statistical significance was set at *p* < 0.05. The analysis was performed using IBM SPSS Statistics for Mac OS, Version 28.0 (IBM Corp., Armonk, NY, USA) licensed to staff of the University of Malaga.

## 5. Conclusions

In summary, this pilot study provides preliminary evidence that the initiation of TNF inhibitor therapy in biologic-naive patients with active RA may be associated with increases in lean mass and improved inflammatory profiles over six months. In addition, our findings show that patients with RA and moderate-to-high inflammatory activity have lower lean mass than healthy individuals. However, due to the exploratory nature of this study and the absence of an untreated RA comparator group, no definitive conclusions regarding the causal effect of TNF inhibitors can be drawn. These results should be interpreted as hypothesis-generating and warrant confirmation in larger, controlled studies.

## Figures and Tables

**Table 1:** Baseline characteristics of 70 patients with moderate–severe RA and 70 controls.

Variable	RA n = 70	Controls n = 70	*p*-Value
Epidemiological			
Female sex, n (%)	57 (81.4)	57 (81.4)	1.000
Age, years, mean (SD)	56.2 (12.3)	54.6 (17.6)	0.528
White race, n (%)	70 (100)	71 (100)	1.000
Comorbid conditions			
Any, n (%)	70 (100.0)	45 (64.3)	<0.001
No. of comorbid conditions, median (IQR)	2.0 (1.0–3.0)	1.0 (0.0–2.0)	0.021
Multimorbidity, n (%)	38 (54.3)	26 (37.1)	0.042
Charlson Comorbidity Index, median (IQR)	1.0 (1.0–2.0)	0.0 (0.0–0.0)	<0.001
Age-adjusted Charlson Comorbidity Index, median (IQR)	2.0 (1.7–3.0)	1.0 (0.7–2.0)	<0.001
Dyslipidemia, n (%)	16 (22.9)	13 (18.6)	0.532
Arterial hypertension, n (%)	20 (28.6)	18 (25.7)	0.704
Smoking			0.037
Nonsmoker, n (%)	31 (44.3)	46 (65.7)	
Exsmoker, n (%)	21 (30.0)	12 (17.1)	
Smoker, n (%)	18 (25.7)	12 (17.1)	
Obesity, n (%)	20 (28.6)	14 (20.0)	0.237
Diabetes mellitus, n (%)	8 (11.4)	5 (7.1)	0.382
Osteoporosis, n (%)	12 (17.1)	9 (12.9)	0.478
Variables associated with RA			
Disease duration, median (IQR), months	76.0 (34.6–184.8)	-	-
Diagnostic delay, median (IQR), months	7.0 (3.9–11.52)	-	-
Erosions, n (%)	35 (50.0)	-	-
RF positive (>10 U/mL), n (%)	60 (85.7)	0 (0.0)	<0.001
**ACPA positive (>20 U/mL), n (%)**	56 (80.0)	0 (0.0)	<0.001
**ACPA high > 340 U/mL, n (%)**	21 (30.0)	0 (0.0)	<0.001
DAS28-CRP, mean (SD)	4.9 (1.15)	-	-
DAS28-CRP, cumulative, mean (SD)	3.7 (0.9)	-	-
HAQ, mean (SD)	1.4 (0.7)	-	-
HAQ, cumulative, mean (SD)	1.0 (0.5)	-	-
Treatment			
csDMARDs, n (%)	70 (100.0)	-	-
Methotrexate, n (%)	45 (64.3)	-	-
Hydroxychloroquine, n (%)	11 (15.7)	-	-
Leflunomide, n (%)	11 (15.7)	-	-
Sulfasalazine, n (%)	19 (27.1)	-	-
Corticosteroids, mg/d, mean (SD)	5.0 (0.0–7.5)	0.0 (0.0–0.0)	
Corticosteroids, n (%)	52 (74.3)	0 (0.0)	

Abbreviations: RA: rheumatoid arthritis; SD: standard deviation; IQR: interquartile range; RF: rheumatoid factor; ACPA: anti-citrullinated peptide antibody; DAS28: 28-joint Disease Activity Score; HAQ: Health Assessment Questionnaire; CRP: C-reactive protein; csDMARD: conventional synthetic disease-modifying antirheumatic drug.

**Table 2:** Characteristics of body composition and inflammation in 70 patients with moderate–severe RA and 70 controls.

Variable	RA n = 70	Controls n = 70	*p*-Value
Anthropometric characteristics			
BMI, kg/m^2^, mean (SD)	27.8 (4.7)	26.9 (4.6)	0.545
Classification of BMI (WHO)			0.122
Low weight (BMI < 18.5), n (%)	0 (0.0)	0 (0.0)	
Normal weight (BMI 18.5–24.9), n (%)	19 (27.1)	27 (38.6)	
Overweight (BMI 25.0–29.9), n (%)	32 (45.7)	33 (47.1)	
Obesity, Grade I (BMI 30.0–34.5), n (%)	15 (21.4)	8 (11.4)	
Obesity, Grade II (BMI 35.0–39.9), n (%)	4 (5.7)	1 (1.4)	
Obesity, Grade III (BMI ≥ 40), n (%)	0 (0.0)	1 (1.4)	
Waist circumference, cm, median (IQR)	96.5 (86.5–105.7)	89.5 (86.0–100.0)	0.080
Hip circumference, cm, mean (SD)	107.1 (9.4)	103.5 (8.7)	0.096
Waist–hip index, mean (SD)	0.9 (0.1)	0.8 (0.1)	0.383
Body composition by DXA			
Total fat mass, kg, mean (SD)	29.1 (9.4)	28.3 (12.2)	0.313
FMI, kg/m^2^, mean (SD)	11.3 (3.5)	11.2 (4.4)	0.900
Total lean mass, kg, mean (SD)	38.0 (8.0)	41.1 (7.7)	0.012
LMI, kg/m^2^, mean (SD)	14.4 (2.3)	15.9 (2.1)	<0.001
RSMI, kg/m^2^, median (IQR)	6.1 (5.6–6.9)	6.4 (5.9–7.3)	0.125
Fat mass arms, kg, mean (SD)	2.8 (1.2)	2.7 (1.0)	0.897
Fat mass legs, kg, mean (SD)	9.9 (3.9)	9.7 (4.3)	0.822
Fat mass trunk, kg, mean (SD)	14.5 (5.3)	14.0 (7.8)	0.729
Fat mass, android area, kg, median (IQR)	2.5 (1.8–3.1)	2.4 (1.8–3.2)	0.740
Fat mass, gynoid area, kg, median (IQR)	5.2 (3.8–6.0)	4.9 (3.7–5.9)	0.746
Lean mass arms, kg, mean (SD)	3.9 (1.0)	4.1 (1.1)	0.250
Lean mass legs, kg, mean (SD)	12.5 (2.6)	12.8 (2.7)	0.511
Lean mass trunk, kg, median (IQR)	19.5 (16.7–21.7)	19.8 (17.3–22.4)	0.636
Lean mass, android area, kg, median (IQR)	2.9 (2.5–3.4)	2.9 (2.6–3.5)	0.813
Lean mass, gynoid area, kg, mean (SD)	5.6 (1.4)	5.8 (1.3)	0.569
Sarcopenia, n (%)	20 (28.6)	9 (12.9)	0.022
Sarcopenic obesity, n (%)	7 (10.0)	1 (1.4)	0.029
Sarcopenic osteoporosis, n (%)	7 (10.0)	3 (4.3)	0.189
Physical activity and Mediterranean diet			
Activity by IPAQ, METs, median (IQR)	594.0 (231.0–1386.0)	990.0 (561.0–1993.5)	0.033
MEDAS (>9), n (%)	50 (71.4)	60 (85.7)	0.039
**Adipokines** and interleukins			
Leptin, ng/mL, median (IQR)	159,283.7 (88,333.0–259,663.4)	164,906.0 (82,963.6–315,133.2)	0.518
Resistin, ng/mL, median (IQR)	5263.5 (3613.2–9461.3)	4587.6 (3182.0–6737.8)	0.135
Adiponectin, µg/mL, median (IQR)	31,555.8 (19,317.3–88,628.1)	19,468.0 (11,361.9–42,551.7)	0.002
IL-6, pg/mL, median (IQR)	5.4 (2.2–12.3)	1.5 (0.9–2.5)	<0.001
CRP, mg/L, median (IQR)	9.4 (4.0–17.2)	3.0 (2.0–4.0)	<0.001
IL-1β, pg/mL, median (IQR)	8.2 (2.9–13.2)	6.2 (2.3–12.3)	0.402
IGF-1, pg/mL, mean (SD)	127,919.9 (20,706.6)	124,325.3 (15,991.6)	0.252
**LDL-oxidase, IU/mL, median (IQR)**	85.2 (65.5–162.2)	62.4 (49.9–75.1)	<0.001
Laboratory data			
ESR, mm/h, median (IQR)	24.0 (13.2–38.0)	10.0 (6.5–16.5)	<0.001
Hemoglobin, g/dL, mean (SD)	12.8 (1.4)	13.4 (1.2)	0.008
Total cholesterol, mg/dL, mean (SD)	189.6 (33.7)	190.5 (35.1)	0.870
LDL cholesterol, mg/dL, mean (SD)	105.5 (28.1)	110.6 (27.3)	0.403
HDL cholesterol, mg/dL, mean (SD)	60.8 (17.4)	59.7 (17.0)	0.771
Triglycerides, mg/dL, median (IQR)	95.0 (71.5–125.0)	94.0 (72.0–151.5)	0.470

Abbreviations: RA: rheumatoid arthritis; SD: standard deviation; IQR: interquartile range; BMI: body mass index; DXA: dual-energy X-ray absorptiometry; FMI: fat mass index; LMI: lean mass index; RSMI: relative skeletal mass index; IPAQ: International Physical Activity Questionnaire; METs: metabolic equivalent of task units; MEDAS, Mediterranean Diet Adherence Screener; IL-6: interleukin 6; CRP: C-reactive protein; ESR: erythrocyte sedimentation rate; IL-1β: interleukin 1 beta; IGF-1: insulin-like growth factor 1; LDL: low-density lipoprotein; HDL: high-density lipoprotein.

**Table 3:** Clinical, body composition, and inflammation-related characteristics of patients at baseline and at 6 months.

Variable	Baseline	6 Months	*p*-Value
Clinical characteristics			
DAS28-CRP, mean (SD)	4.8 (1.1)	3.1 (1.2)	<0.001
NTJ, median (IQR)	5.5 (2.0–10.0)	1.0 (0.0–2.0)	<0.001
NSJ, median (IQR)	3.0 (1.0–6.0)	0.0 (0.0–1.0)	<0.001
**VAS general, mm, median (IQR)**	70 (60–90)	10 (5–50)	<0.001
VAS pain, mm, median (IQR)	70 (60–90)	7 (2–50)	<0.001
VAS physician, mm, median (IQR)	70 (60–80)	5 (2–30)	<0.001
HAQ, mean (SD)	1.3 (0.6)	1.0 (0.6)	<0.001
Anthropometric characteristics			
BMI, kg/m^2^, mean (SD)	27.8 (4.7)	27.4 (5.2)	0.403
Waist circumference, cm, mean (SD)	95.2 (12.3)	94.9 (10.2)	0.760
Hip circumference, cm, mean (SD)	103.8 (8.7)	102.2 (6.7)	0.690
Waist–hip ratio, mean (SD)	0.9 (0.1)	0.9 (0.1)	0.880
Body composition by DXA			
Total fat mass, kg, mean (SD)	29.1 (9.4)	26.8 (9.5)	0.121
FMI, kg/m^2^, mean (SD)	11.3 (3.5)	10.3 (3.5)	0.243
Lean mass total, kg, mean (SD)	38.0 (8.0)	40.2 (10.5)	<0.001
LMI, kg/m^2^, mean (SD)	14.4 (2.3)	15.3 (3.0)	0.016
RSMI, kg/m^2^, median, (p25-p75)	6.1 (5.6–6.9)	6.2 (5.8–7.1)	<0.001
Fat mass, arms, kg, mean (SD)	2.8 (1.2)	2.6 (0.9)	0.752
Fat mass, legs, kg, mean (SD)	9.9 (3.9)	9.5 (3.6)	0.497
Fat mass, trunk, kg, mean (SD)	14.5 (5.3)	13.8 (5.7)	0.292
Fat mass, android area, kg, median (IQR)	2.5 (1.8–3.1)	2.4 (1.6–3.1)	0.299
Fat mass, gynoid area, kg, median (IQR)	5.2 (3.8–6.0)	5.0 (3.7–5.7)	0.815
Lean mass, arms, kg, mean (SD)	3.9 (1.0)	4.1 (1.2)	<0.001
Lean mass, legs, kg, mean (SD)	12.5 (2.6)	12.8 (3.3)	0.468
Lean mass, trunk, kg, median (IQR)	19.5 (16.7–21.7)	19.8 (17.4–22.5)	0.003
Lean mass, android area, kg, median (IQR)	2.9 (2.5–3.4)	2.9 (2.5–3.3)	0.116
Lean mass, gynoid area, kg, mean (SD)	5.6 (1.4)	5.9 (1.3)	0.031
Sarcopenia, n (%)	20 (28.6)	8 (11.4)	0.004
Sarcopenic obesity, n (%)	7 (10.0)	2 (2.9)	0.056
Sarcopenic osteoporosis, n (%)	6 (8.6)	5 (7.0)	0.708
Physical activity and Mediterranean diet			
IPAQ activity, METs, median (IQR)	594.0 (231.0–1386.0)	946.5 (358.8–1640.2)	0.0450
MEDAS (>9), n (%)	50 (71.4)	51 (72.9)	0.784
**Adipokines** and interleukins			
Leptin, ng/mL, median (IQR) *	159,283.7 (88,333.0–259,663.4)	19,541.4 (7794.5–29,933.4)	<0.001
Resistin, ng/mL, median (IQR)	5263.5 (3613.2–9461.3)	11,076.7 (7187.8–16,763.9)	<0.001
Adiponectin, µg/mL, median (IQR)	31,555.8 (19,317.3–88,628.1)	50,807.0 (23,635.0–91,249.7)	0.035
IL-6, pg/mL, median (IQR)	5.4 (2.2–12.3)	2.2 (1.0–6.4)	<0.001
PCR, mg/L, median (IQR)	9.4 (4.0–17.2)	1.9 (2.0–8.1)	<0.001
IL-1β, pg/mL, median (IQR)	8.2 (2.9–13.2)	3.1 (0.8–9.1)	<0.001
IGF-1, pg/mL, mean (SD)	127,919.9 (20,706.6)	137,568.6 (20,867.4)	<0.001
**LDL-oxidase, IU/mL, median (IQR)**	85.2 (65.5–162.2)	75.1 (54.3–131.2)	<0.001
Laboratory data			
ESR, mm/h, median (IQR)	24.0 (13.2–38.0)	18.5 (9.7–31.2)	0.023
Hemoglobin, g/dL, mean (SD)	12.8 (1.4)	13.0 (1.3)	0.029
Total cholesterol, mg/dL, mean (SD)	190.5 (33.7)	191.0 (35.0)	0.874
LDL cholesterol, mg/dL, mean (SD)	110.0 (29.1)	117.1 (34.1)	0.191
HDL cholesterol, mg/dL, mean (SD)	63.1 (16.1)	61.6 (17.8)	0.364
Triglycerides, mg/dL, median (IQR)	95.0 (71.5–125.0)	91.0 (67.0–127.0)	0.997

Abbreviations: SD: standard deviation; IQR: interquartile range; BMI: body mass index; VAS: visual analog scale; FMI: fat mass index; LMI: lean mass index; RSMI: relative skeletal mass index; IPAQ: International Physical Activity Questionnaire; METs: metabolic equivalent of task units; MEDAS, Mediterranean Diet Adherence Screener; IL-6: interleukin 6; CRP: C-reactive protein; ESR: erythrocyte sedimentation rate; IL-1β: interleukin 1 beta; TNF-α: tumor necrosis factor alpha; IGF-1: insulin-like growth factor 1; LDL: low-density lipoprotein; HDL: high-density lipoprotein.

**Table 4:** Correlation between FFMI, total lean mass, and increase in lean mass with TNF inhibitors, together with clinical characteristics of patients with RA.

Variable	FFMI *p*-Pearson	Lean Mass *p*-Pearson	∆ Lean Mass *p*-Pearson
Age, years	−0.052	−0.135	−0.103
Disease duration, months	0.140	0.054	−0.400 *
Diagnostic delay, months	−0.094	−0.024	0.164
BMI, kg/m^2^	0.505 **	0.314 *	−0.019
RF (U/mL)	0.008	0.053	−0.045
ACPA (U/mL)	−0.035	−0.084	0.059
DAS28-CRP	−0.127	−0.014	−0.318 *
Cumulative DAS28-CRP	−0.198	−0.099	−0.357 *
HAQ	0.017	0.085	−0.323 *
Cumulative HAQ	0.022	−0.004	−0.343 *
Activity according to IPAQ (METs)	−0.041	−0.020	−0.120
Leptin, ng/mL	0.105	−0.055	−0.174
Resistin, ng/mL	−0.093	−0.017	0.026
Adiponectin, µg/mL	−0.294 *	−0.280 *	0.126
IL-6, pg/mL	−0.389 **	−0.285 *	−0.060
CRP, mg/L	−0.234 *	−0.040	−0.349 *
IL-1β, pg/mL	0.105	0.173	−0.061
IGF-1, pg/mL	−0.310 *	−0.379 *	−0.231
LDL-oxidase, IU/mL	−0.070	−0.117	−0.053
ESR, mm/h	−0.377 **	−0.237	0.063
Hemoglobin, g/dL	0.187	0.206	−0.007

Abbreviations: FFMI: fat-free mass index; RA: rheumatoid arthritis; RF: rheumatoid factor; ACPA: anti-citrullinated peptide antibody; DAS28: 28-joint Disease Activity Score; HAQ: Health Assessment Questionnaire; BMI: body mass index; IPAQ: International Physical Activity Questionnaire; METs: metabolic equivalent of task unit; IL-6: interleukin 6; CRP: C-reactive protein; IL-1β: interleukin 1 beta; IGF-1: insulin-like growth factor 1; ESR: erythrocyte sedimentation rate; LDL: low-density lipoprotein. * *p* < 0.05; ** *p* < 0.001.

**Table 5:** Univariate and multivariate analyses of the characteristics associated with baseline lean mass in patients with rheumatoid arthritis.

Predictor	Univariate		Multivariate		
	B	95% CI	B	95% CI	*p*-Value
LMI *					
Female sex	−2.472	−3.848, −1.097	−3.299	−4.662, −1.936	<0.001
Age, years	0.005	−0.043, 0.054			
BMI	0.270	0.151, 0.389	0.300	0.105, 0.416	<0.001
Cumulative DAS28	−0.278	−0.914, 0.358			
Cumulative HAQ	0.846	−0.035, 1.728			
CRP, mg/L	−0.066	−0.090, −0.039	−0.063	−0.096, −0.030	0.001
Adiponectin	−1.632	−3.074, −0.191			
Leptin	1.352	0.145, 3.849			
METs	0.220	−1.519, 1.958			
Lean mass **					
Female sex	−13.115	−17.088, −9.141	−13.288	−17.598, −8.978	<0.001
Age, years	−0.088	−0.253, 0.078			
BMI	0.575	0.126, 1.025	0.560	0.186, 0.934	0.004
Cumulative DAS28	−0.837	−3.034, 1.360			
Cumulative HAQ	1.019	−2.093, 4.131			
CRP, mg/L	−0.098	−0.214, −0.018	−0.151	−0.258, −0.044	0.007
Adiponectin	−6.679	−11.575, 1.783			
Leptin	−1.519	−6.657, 3.619			
METs	1.112	−4.333, 6.557			
∆ lean mass ¥					
Female sex	−3.891	−8.405, 0.623			
Age, years	−0.060	−0.225, 0.106			
BMI	−0.031	−0.500, 0.437			
Cumulative DAS28	−2.295	−4.179, −0.410	−2.578	−4.963, −0.193	0.035
Cumulative HAQ	−3.343	−6.218, −0.469			
CRP, mg/L	−0.153	−0.271, −0.035	−0.198	−0.322, −0.074	0.003
Adiponectin	−1.144	−7.165, 4.877			
Leptin	−3.624	−8.077, 0.828			
METs	−1.144	−7.165, 4.877			

* Adjusted R2 = 0.524. ** Adjusted R2 = 0.486. ¥ Adjusted R2 = 0.333. Abbreviations: LMI: lean mass index; BMI: body mass index; DAS28: 28-joint Disease Activity Score; HAQ: Health Assessment Questionnaire; CRP: C-reactive protein; METs: metabolic equivalent of task units. Variables included in the equation: sex, age, BMI, cumulative DAS28, cumulative HAQ, CRP, adiponectin, leptin, and METs.

## Data Availability

Data is contained within the article.

## Abbreviations

The following abbreviations are used in this manuscript:

RA	rheumatoid arthritis
RF	rheumatoid factor
ACPA	anti-citrullinated peptide antibody
DAS28	28-joint Disease Activity Score
CRP	C-reactive protein
HAQ	health Assessment Questionnaire
IL	interleukin
DMARD	disease-modifying antirheumatic drug
BMI	body mass index
DXA	dual-energy X-ray absorptiometry
FMI	fat mass index
FFMI	fat-free mass index
LMI	lean mass index
RSMI	relative skeletal mass index
